# Plasticity to drought and ecotypic differentiation in populations of a crop wild relative

**DOI:** 10.1093/aobpla/plaa006

**Published:** 2020-02-12

**Authors:** S Matesanz, M Ramos-Muñoz, B Moncalvillo, M L Rubio Teso, S L García de Dionisio, J Romero, J M Iriondo

**Affiliations:** Área de Biodiversidad y Conservación, Universidad Rey Juan Carlos, C/Tulipán s/n, Móstoles, Madrid, Spain

**Keywords:** Adaptive divergence, cogradient variation, common garden, phenotypic plasticity, populations, water stress

## Abstract

Populations of widely distributed species often exhibit geographic variation in functional traits in response to environmental heterogeneity. Such trait variation may be the result of different adaptive mechanisms, including genetically based differentiation, phenotypic plasticity or a combination of both. Disentangling the genetic and environmental components of trait variation may be particularly interesting in crop wild relatives, since they may provide unique reservoirs of genetic diversity for crop improvement. In this study, we assessed ecotypic differentiation and patterns of plasticity to drought in populations of *Lupinus angustifolius*, a Mediterranean crop wild relative, from two climatically distinct regions in the Iberian Peninsula. Using an outdoor common garden, we compared phenotypic responses of inbred maternal families to two ecologically meaningful water availability treatments (drought and high-moisture). We measured 18 different functional traits related to growth, morphology, phenology and reproduction. Plants in the drought treatment grew less, had lower leaf chlorophyll content and photochemical efficiency, but also reproduced faster, produced larger seeds and altered leaflet morphology through increased leaflet thickness, higher leaflet dry matter content and lower specific leaf area. We also found significant differences between regions that likely reflect adaptation to climatically distinct environments, with populations from the south showing a faster onset of reproduction, higher leaf thickness and higher seed size, consistent with the drier conditions experienced in southern sites. Plasticity to drought was in most cases in the same direction as quantitative genetic differentiation (i.e. cogradient variation), providing evidence of the adaptive value of the plastic change. Our results show that both genetic differentiation and plasticity can generate adaptive phenotypic variation in *L. angustifolius*, and help to identify potentially valuable genetic resources to incorporate into breeding programmes.

## Teaser

We assessed adaptive differentiation and phenotypic plasticity in populations of *Lupinus angustifolius*, a Mediterranean crop wild relative, from two regions in the Iberian Peninsula. We compared how plants from different populations responded to two water availability treatments, drought and high-moisture, and measured several functional traits. Plants in the drought treatment reproduced faster, produced larger seeds and showed changes in leaf morphology, consistent with adaptive plasticity. Significant differences between regions likely reflect adaptation of the southern populations to their drier environment. We conclude that both genetic differentiation and plasticity can contribute to the adaptation of *L. angustifolius* to different water availability environments, and help to identify valuable genetic resources for breeding.

## Introduction

Plant functional traits often vary across space, particularly when species encounter contrasting environmental conditions throughout their range. Such phenotypic variation is determined by both genetic and environmental effects. Understanding their relative contribution to trait variation and how they influence plant adaptation are central questions in evolutionary ecology (Mitchell-Olds *et al.* 2007; Albert *et al.* 2011). In the classical Darwinian paradigm, plants accommodate environmental variation primarily through natural selection. Differential selection pressures across contrasting conditions drive the evolution of functional traits, resulting in genetic differentiation among populations and the formation of ecotypes (Endler 1986). However, it is now well-established that phenotypic plasticity, the ability of a genotype to alter its phenotype in response to environmental variation, constitutes a second, yet equally important, mode of adaptation in plants (Sultan 1995; Pigliucci 2001 and references therein).

Not all plastic responses can be assumed to be adaptive, because they include both unavoidable effects of resource limitations on different aspects of the phenotype, and active adjustments that boost individual performance in the triggering environment (Sultan 2000; Nicotra *et al.* 2010). However, the adaptive responses—which allow to maintain function across diverse conditions—are those of particular relevance from an evolutionary perspective. Despite their importance, determining the adaptive value of plastic responses is not straightforward (see e.g. Arnold *et al*. 2019), and many studies base their interpretation on whether plastic responses to stress match similar adaptations favoured by selection in a given environment (Sultan 1995; Gianoli and González-Teuber 2005; Matesanz *et al.* 2012; Cavender-Bares and Ramírez-Valiente 2017).

Adaptive genetic differentiation and phenotypic plasticity are often intimately related mechanisms. For instance, ecotypic differentiation may be obviated if individuals are sufficiently plastic to produce appropriate phenotypes in contrasting conditions (Sultan and Spencer 2002; Crispo 2008; Baythavong 2011; Matesanz and Valladares 2014 and references therein). Plasticity and genetic divergence could thus be considered mutually exclusive adaptive mechanisms. Alternatively, genetic and environmental effects on traits may co-vary such that the plastic change is in the direction of the genetic differentiation among populations (i.e. cogradient variation; Levins 1968; Conover and Schultz 1995; Eckhart *et al.* 2004; Crispo 2008). For example, in populations established across gradients of water availability, selection may favour trait values that enhance drought tolerance in the driest sites (e.g. early flowering, higher seed mass, etc.), resulting in adaptive population differentiation. Plasticity to lower water availability may produce trait shifts that match those shaped by selection (i.e. advanced flowering, increased seed mass, etc.) in response to water stress. In these cases, ecotypic differences and adaptive plasticity may be complementary rather than mutually exclusive ways to accommodate environmental variability in plants (Sultan 1995; Crispo 2008; Matesanz *et al.* 2010).

To investigate population genetic differentiation and plasticity patterns, common gardens that simulate variations of relevant environmental factors according to natural variation are particularly useful, as they allow the identification of the precise factors driving phenotypic differentiation (Franks *et al.* 2014; Matesanz and Ramírez-Valiente 2019). When replicated at the genotype (or family) level, these experiments may also provide evidence of quantitative genetic variation within populations, which constitutes the substrate for further evolution. Finally, because both genetic differentiation and plasticity are simultaneously assessed, these experiments allow to evaluate whether these mechanisms constitute alternative or complementary modes of plant adaptation.

In this study, we assessed population differentiation and patterns of plasticity to drought in populations of *Lupinus angustifolius*, a crop wild relative of Mediterranean distribution. Crop wild relatives include both crop progenitors as well as species related to them, providing unique reservoirs of genetic diversity that may be useful for crop improvement (Hajjar and Hodgkin 2007; Heywood *et al.* 2007; Dwivedi *et al*. 2008). For instance, populations of these species may contain specific adaptations to environmental factors experienced in natural conditions that are desirable for agriculture (Hajjar and Hodgkin 2007), which may emerge both as genetic differentiation in functional traits or as the plasticity of those traits (Matesanz and Milla 2018). Therefore, crop wild relatives constitute excellent models to assess the genetic and environmental sources of phenotypic variation.

Throughout its wide natural distribution, populations of *L. angustifolius* occur along vast temperature and drought gradients (Berger *et al.* 2017). We focused on genetic differentiation and plasticity patterns in response to variation in water availability, because drought is considered the main constraint to plant growth and performance in the Mediterranean region (Blondel *et al.* 2010; Matesanz and Valladares 2014). Using an outdoor common garden, we compared phenotypic responses to two ecologically meaningful water availability treatments in inbred maternal families of populations from two climatically distinct regions in the Iberian Peninsula. The assessment of the precise patterns of phenotypic diversification of wild relatives may be particularly useful in crops with a recent and fragmented history of domestication such as the narrow-leafed lupin, where only a small fraction of the genetic diversity of the species has been retained during domestication (Berger *et al.* 2013). We measured a suite of life-history, functional and reproductive traits to address the following specific questions: (i) What is the effect of water stress on the phenotypic response of populations? (ii) Is there ecotypic differentiation evidenced by genetically based differences between regions? (iii) Are plastic changes in the same direction as those found in regional differentiation, evidencing cogradient variation? We discuss our findings in the context of crop improvement and the identification of potentially useful genetic resources.

## Materials and Methods

### Study species and population sampling

**Figure 1.**
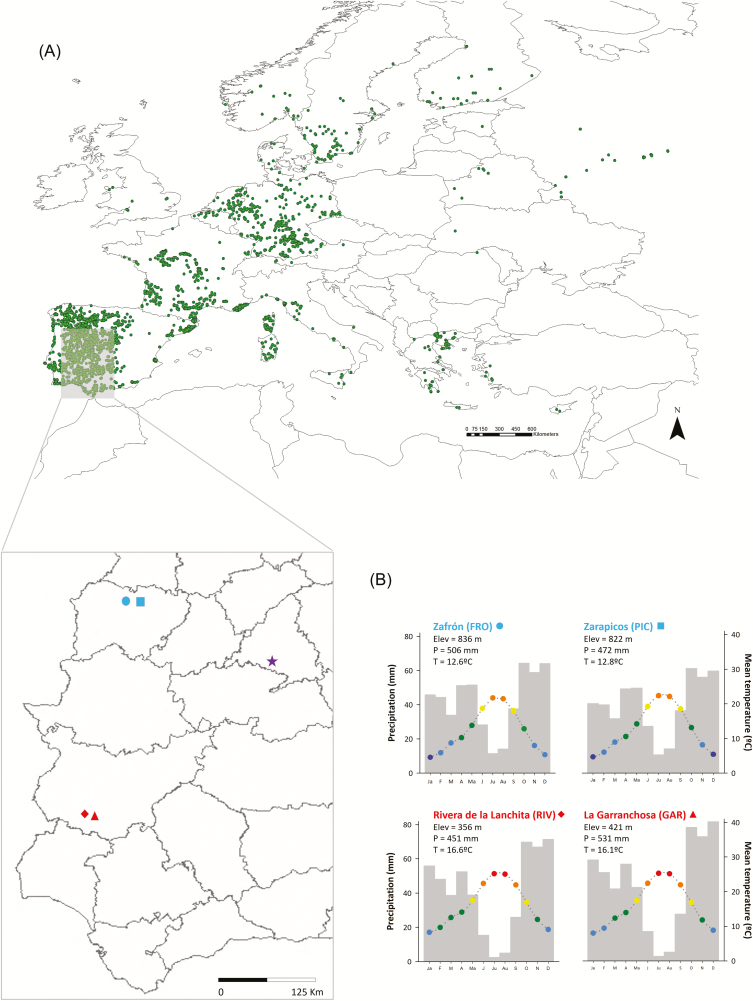
(A) Natural distribution of *Lupinus angustifolius*; each dot corresponds to records of presence of the species (source: GBIF.org (27 November 2018) GBIF Occurrence Download https://doi.org/10.15468/dl.4ka9lc). The location of the two northern (blue) and southern (red) populations of the species in the Iberian Peninsula, and of the common garden (purple star) is presented in the enlarged area; (B) climographs showing monthly precipitation and mean temperature, based on climatic data from 1979 to 2013 (see text for details). Note that the precipitation range is equal to twice the temperature scale. Elevation, mean annual precipitation and temperature are also shown for each population. Each colour in the temperature graph represents a 5 °C range (purple: 0–5 °C; blue: 5–10 °C; green: 10–15 °C; yellow: 15–20 °C; orange: 20–25 °C and red: 25–30 °C).

The narrow-leafed lupin or blue lupin (*L. angustifolius*) is an annual herb widespread throughout the Mediterranean Basin and introduced as a livestock-feeding crop in many parts of the world, including Australia and northern Europe (Fig. 1). In natural conditions, it occurs in disturbance-prone environments, including roadsides and abandoned croplands, inhabiting well-drained, acid or neutral soils. It can reach up to 100 cm in height, with long inflorescences of up to 30 purple-blue flowers, and has compound leaves of 5–9 narrow leaflets (Capon *et al.* 2009). The species mainly self-pollinates, producing pods of 3–6 seeds.

**Figure 2.**
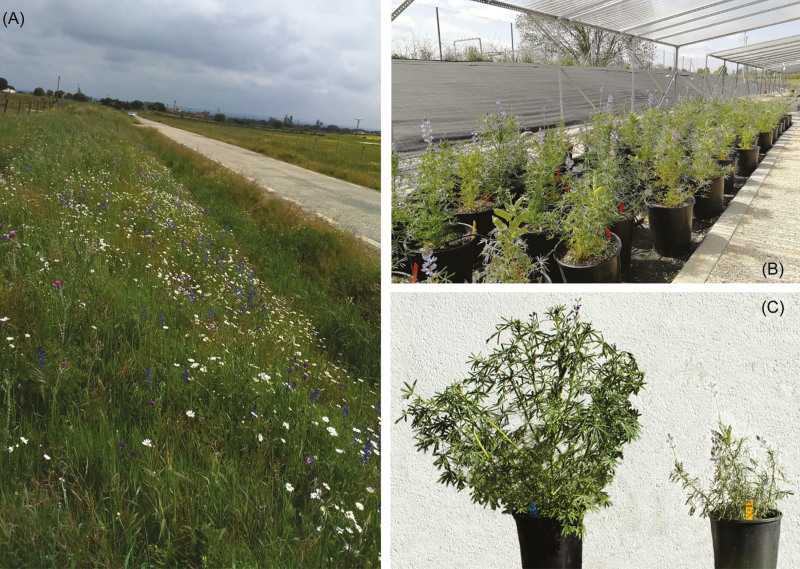
(A) View of one of the northern populations, Zafrón (FRO); (B) plants growing below the rain exclusion structures used to implement the watering treatments in the plasticity to drought experiment; (C) Full-siblings from the same *Lupinus angustifolius* family grown in high-moisture (left), and drought (right) watering treatments. Phenotypic differences among these genetically uniform individuals include changes to phenology, physiology, leaf structure and reproduction.

Four populations of the species were selected in two geographically and climatically contrasting regions (Figs 1 and 2A; **see****Supporting Information—Table S1**; hereafter northern and southern populations). Distance between populations within the same region ranged between 12 and 20 km, and distance among regions was >300 km. Long-term climatic data for each population site (1979–2013; Karger *et al.* 2017) indicate similar annual precipitation levels across populations but strong differences in annual temperature: the mean, minimum and maximum temperatures in the two northern populations are 3.5–4.0 °C colder than in the southern populations (**see****Supporting Information—Table S1**; Fig. 1B). Such strong differences in temperature, despite similar precipitation, result in substantially different aridity levels. According to the Lang index of aridity (Lang 1920), the two northern populations occur in subhumid climate and the southern populations occur in semi-arid climates **[see****Supporting Information—Table S1****]**. The sampled populations occurred in roadsides and were composed of hundreds of individuals (Fig. 2A). In the spring of 2016, mature pods of >100 maternal plants separated at least 1 m from each other were collected, bagged and transported to the lab.

### Experimental set-up and conditions in the plasticity experiment

The experiment was performed in the CULTIVE facilities at URJC. In November 2016, three field-collected seeds from each maternal plant were scarified and sown. Seedlings were raised to maturity in uniform, favourable conditions to remove environmentally induced maternal effects from field-collected seeds, and to produce inbred families (full-siblings). Pots were maintained in a greenhouse till March 2017 and subsequently moved outdoors. They were maintained at field capacity throughout the entire plant’s cycle. The seeds collected in this generation were used in the plasticity experiment.

In November 2017, we randomly selected 21 maternal plants per population. Sixteen seeds per maternal plant (hereafter families) were individually weighed in a Mettler Toledo MX5 microbalance (1 µg precision; Mettler Toledo, Columbus, OH, USA) to obtain a family-level average seed size **[see****Supporting Information—Fig. S1****]**. Twelve seeds per maternal plant were later scarified to promote germination and were sown in 0.5-L pots (two seeds per pot) containing a 2:1 mixture of topsoil and sand. Pots were randomly thinned to one seedling after 3 weeks. Pots were randomly arranged in a hoop greenhouse and kept for 3 months, where soil moisture was maintained at field capacity (see environmental conditions for this period in **Supporting Information—Fig. S2**). The final size of the experiment was 504 plants (4 populations × 21 families per population × 6 plants per family).

In February 2018, plants were individually transplanted into 6.4-L plastic pots (20 cm × 22.5 cm) filled with the same substrate as above and moved from the greenhouse to the outdoors cultivation facility. Long-term climatic conditions in this location fall within the range of those experienced by the species in field conditions **[see****Supporting Information—Table S1****]**, creating a natural environment for our experiment. Soil moisture in pots was maintained at field capacity for several weeks to allow acclimation to outdoors conditions. In March 2018, three randomly selected plants per family were assigned to each of two experimental treatments of contrasting water availability (high-moisture and drought, see below). The experiment lasted 4 months (March to June) and ended when all plants had completed their cycle.

To ensure the correct implementation of the watering treatments, pots were placed in purposefully built rain exclusion structures that eliminated all natural precipitation (Fig. 2B). The structures were built using iron profiles topped with corrugated transparent polycarbonate sheets (Rooflite, Wetherill Park, Australia). The polycarbonate sheets were assembled on the profiles with a ≈10° inclination, to avoid the accumulation of rainfall on top of the structures. The height of the structures (1.5 m on the shortest side) and the use of transparent roof material guaranteed a minimal effect of the structures on the conditions experienced by the plants compared to outdoors (data not shown). We established below the structures a HOBO Micro station data logger (H21-USB, Onset Co., Bourna, MA, USA) that recorded photosynthetic active radiation (PAR), relative humidity and air temperature every 10 min throughout the experiment **[see****Supporting Information—Fig. S3****]**.

We implemented two contrasting and realistic watering treatments that reflect the soil moisture variation occurring in natural conditions: high-moisture and drought. In the high-moisture treatment, plants were kept at field capacity (20–25 % soil water content, SWC, for our soil mix; **see****Supporting Information—Fig. S4**). In the drought treatment, SWC was progressively reduced in the pots to simulate the soil drying process occurring in the field, and pots were then kept at 35–40 % of field capacity (7–8 % SWC; **see****Supporting Information—Fig. S4**). The watering treatments were implemented by modifying the number and duration of watering events. In each of them, drip irrigation was applied on a pot-level basis by pressure-compensating drippers (Rainbird XB PC 05; Rainbird Iberica, Madrid, Spain; Fig. 1C). To monitor the successful implementation of the watering treatments, SWC was measured every 3–4 days in 15–20 pots per treatment, using a HH2 Moisture Meter (Delta-T Devices, Cambridge, UK; **see****Supporting Information—Fig. S4**).

### Data collection

#### Growth traits

We measured plant height (length of primary stem) in all plants at the onset and end of the treatments of the plasticity experiment. From these, we calculated relative growth rate as RGR = (Ln *S*_2_ − Ln *S*_1_)/*T*_2−1_, where *S*_1_ and *S*_2_ are plant height at time 1 and 2, respectively, and *T*_2−1_ is the time elapsed between the two measurements. Finally, we collected all the above-ground biomass of each plant once all the reproductive biomass was mature and collected. Since in this species most leaves are shed during pod and seed maturation, post-reproduction standing biomass is mostly composed of leafless shoots.

#### Physiological traits

In May 2018, we measured two traits that are proxies of photosynthetic activity. First, midday photochemical efficiency (*F*_v_/*F*_m_) was measured in all plants with a portable pulse-modulated fluorometer (FMS2, Hansatech, UK). Measurements were taken from 13:00 to 16:00 p.m. (UTC + 2) during three consecutive sunny days. One leaf from a secondary branch was adapted to dark for 30 min with leaf clips to ensure that all PSII centres opened (Maxwell and Johnson 2000). Minimal (*F*_o_) and maximal (*F*_m_) fluorescence were used to calculate photochemical efficiency as *F*_v_/*F*_m_ = (*F*_m_ − *F*_o_)/*F*_m_, where *F*_v_ is the difference between *F*_m_ and *F*_o_. Furthermore, we measured leaf chlorophyll content in one leaf of each plant with a SPAD 502 chlorophyll meter (Konica Minolta, Tokyo, Japan).

#### Morphological traits

In May 2018, eight leaflets per plant were randomly selected. To sample within-plant variability in leaf traits, we collected the centremost leaflet of eight different, fully expanded leaves from secondary branches. They were stored in plastic bags filled with water-saturated filter paper and kept overnight in a fridge at 4 °C to ensure complete hydration of the material. The next morning, leaflets were weighed in a Kern ABJ 120-4M analytical balance (Kern & Sohn GmbH, Albstadt, Germany) to obtain their saturated weight. Then, leaflet thickness was measured in three randomly selected leaflets per plant using a dial thickness gauge (Mitutoyo Co., Japan), and taking two different measurements in each leaflet. Finally, all leaflets were scanned and oven-dried for 48 h at 60 °C. Leaflet area was calculated in Adobe Photoshop (Adobe Systems Inc., California, USA). Specific leaflet area (SLA) was estimated as the ratio of the one-side area of a fresh leaflet divided by its oven-dry mass, and leaflet dry matter content (LDMC) was calculated as leaflet dry weight divided by its saturated weight.

#### Phenology

Flowering phenology was monitored in all plants with 2–3 censuses per week throughout the experiment. We considered three different phenological events: onset of flower bud formation (flower buds visible to the naked eye), onset of flowering (appearance of open flowers) and onset of fruiting (green pods visible to the naked eye). These variables were defined as the number of days elapsed between 1 January and the dates in which the first flower bud, flower and fruit appeared in each plant. The duration of flowering onset was calculated for each population and treatment as the difference between the dates when the first flower was observed in each individual.

#### Reproductive success

We collected all pods of all plants at their peak of maturation (late May to mid-June in the drought treatment and mid-June to early July in the high-moisture treatment), and weighed them in an analytical balance. All other reproductive biomass (undeveloped pods and/or aborted flowers) was also collected at the end of the experiment and weighed to compute the total reproductive biomass per plant. The total number of viable pods per plant was counted. Then, 15 pods per plant were opened to count the number of viable seeds per pod. These pods were selected from the entire reproductive period of each plant and from both primary and secondary branches. The total number of seeds per plant was calculated by multiplying the average number of seeds per pod by the total number of pods. Finally, 10 seeds per plant (when available) were individually weighed in a Mettler Toledo MX5 microbalance.

### Data analyses

To test the effect of the watering treatments on phenotypic traits and whether plastic responses differed among regions and populations, we used linear mixed models with restricted maximum likelihood (REML) on each phenotypic trait. We included region, treatment and their interaction as fixed effects. Family and population nested within region were included as random factors. We fitted the models using function *lmer* (package lme4; Bates *et al.* 2015). Significance of fixed factors was tested using function *Anova* (package car; Fox and Weisberg 2011), with type III sum of squares and the Kenward-Roger approximation to calculate the residual degrees of freedom. To assess differences among regions within watering treatments, the same model (excluding treatment) was ran for each watering treatment. To assess the significance of the population factor, we compared the full model (including fixed and random factors) with the reduced model (dropping the factor population) using function *anova* (package stats) and REML fit. Marginal *R*^2^, representing the variance explained by fixed factors, and conditional *R*^2^, which represents variance explained by both fixed and random factors, were calculated using *r.squaredGLMM* (package MuMIn; Barton 2019).

A significant effect of region indicates genetically based ecotypic phenotypic differentiation; a significant effect of treatment indicates plasticity for the trait, and, finally, a significant region × treatment interaction indicates that plants from different regions respond differently to the watering treatments, i.e. differential plasticity (non-parallel norms of reaction). A significant effect of population indicates differentiation among populations not explained by regional differences. When this was the case, a second model was ran removing region and including population, treatment and their interaction as fixed factors. Differences among populations within treatments were assessed via Tukey *post hoc* tests on marginal means, using function *emmeans* (package emmeans; Lenth 2019) separately for each watering treatment. Differences among regions and treatments in the duration of flowering were assessed using a linear model with region and treatment as fixed factors. Data were square root- or log-transformed when necessary to meet assumptions of normality and homoscedasticity. Data from four plants that were severely damaged or died throughout the experiment were removed from the data set. They were randomly distributed across populations and treatments.

## Results

Phenotypic expression of all functional traits was significantly affected by the watering treatments (treatment effect in Table 1; Fig. 2C). These patterns of plastic response differed significantly between northern and southern populations for several traits (as shown by the significant region × treatment interaction, Table 1). Furthermore, northern and southern populations differed on average for most growth and phenology traits (region effect, Table 1), although we also found differences among populations not explained by region of origin in multiple traits (population effect, Table 1).

**Table 1.** Results of linear mixed models testing the effects of region, treatment and their interaction on functional traits. Family and population (nested in region) were included as random factors. *F*-statistic (χ ^2^ for the random factor) is shown for each term. Significant terms are shown in bold (*P < 0.05; **P < 0.01; ***P < 0.001; ^ǂ^ indicates a marginally significant effect, *P* < 0.1). *R*^2^_m_ = marginal *R*^2^; *R*^2^_c_ = conditional *R*^2^. df = 1 for all fixed terms. See text for details on statistical analyses. Note: italicized terms are significant when false discovery rate correction is applied.

	Region	Treatment	Region × Treatment	Population	*R* ^2^_c_	*R* ^2^_m_
	*F*	*F*	*F*	χ ^2^		
Growth traits						
Initial plant height	20.748*	0.071	0.577	1.658	0.221	0.460
Final plant height	12.456^ǂ^	2718.957***	21.788***	0.001	0.840	0.848
Relative growth rate	38.553*	1431.748***	6.153*	1.439	0.734	0.778
Final aerial biomass	1.686	2580.744***	0.004	4.955*	0.830	0.841
Phenology						
Onset of flower bud formation	139.8754**	11.905***	0.238	2.859	0.664	0.735
Onset of flowering	134.7737**	15.003***	0.338	3.906*	0.716	0.793
Onset of fruit formation	13.0312^ǂ^	45.354***	3.367^*ǂ*^	26.546	0.451	0.639
Physiology						
Chlorophyll content	0.234	138.374***	2.572	12.435***	0.202	0.300
Midday photochemical efficiency	2.155	206.877***	10.243**	1.808	0.304	0.332
Leaf morphology and structure						
Leaf thickness	8.6641^*ǂ*^	99.0975***	0.112	5.388*	0.228	0.289
Leaf dry matter content	3.174	109.289***	12.991***	5.6025*	0.209	0.364
Leaf area	0.008	1619.893***	20.165***	0.255	0.734	0.778
Specific leaf area	0.467	222.6144***	7.314**	25.736***	0.282	0.421
Reproductive traits						
Reproductive biomass	24.759*	274.743***	0.993	2.279	0.423	0.467
Number of fruits	1.900	404.696***	0.720	6.394*	0.436	0.488
Seeds per fruit	4.417	217.299***	29.850***	4.860*	0.316	0.485
Total seeds per plant	1.030	436.290***	0.000	3.929*	0.451	0.495
Individual seed mass	37.089*	26.335***	9.1874**	9.954**	0.537	0.656

### Growth traits

**Figure 3.**
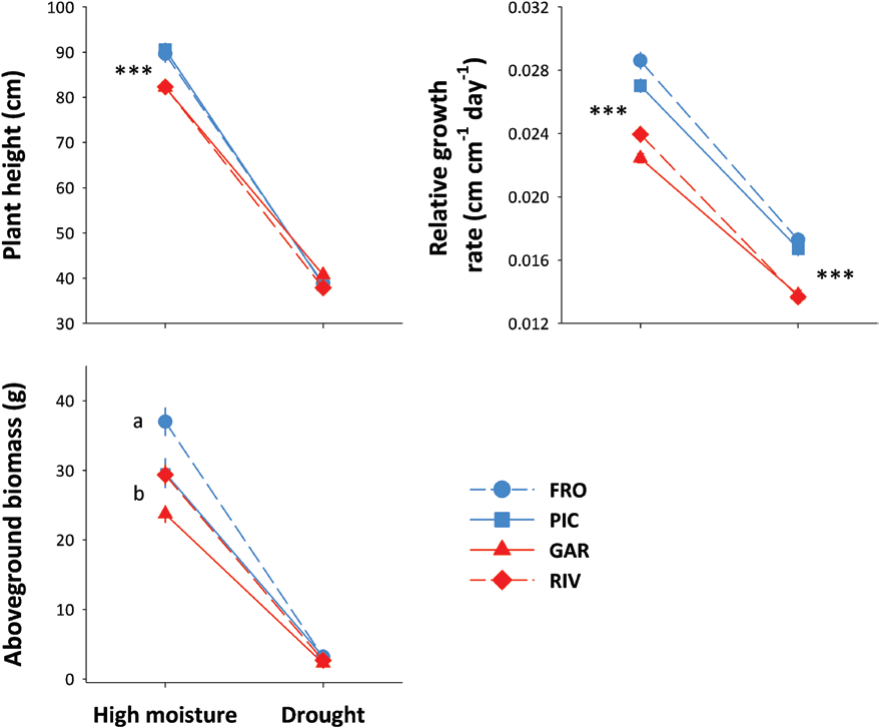
Population differences in growth traits in two contrasting water availability treatments. Each line shows the norm of reaction of each of two northern (blue) and two southern (red) populations for a given trait. Observed population means ± SE are shown for 21 families per population, with three replicates per family and treatment. Significant regional differences in each watering treatment are shown with asterisks (no asterisk: not significant differences; *P < 0.05; **P < 0.01; ***P < 0.001). Different letters indicate significant differences among populations in mean trait values within each watering treatment, according to Tukey *post hoc* tests. *N* = 504 plants for all traits. Population codes are those in Fig. 1.

Plants from all populations grew more, in terms of height and final above-ground biomass, in the high-moisture treatment than plants in the drought treatment (treatment effect, Table 1; Fig. 3). On average, there was a 2-fold difference between treatments in height and relative growth rate, and a 10-fold increase in final biomass in the high-moisture treatment. Although plants from the southern populations were initially taller than those from the northern populations (significant region effect, Table 1), the latter showed higher relative growth rates, particularly in the high-moisture treatment (significant region × treatment, Table 1), leading to similar or even larger plants from the northern sites at the end of the experiment (Fig. 3). A similar pattern was found for the final above-ground biomass, where population (but not regional) differences were observed only in the high-moisture treatment (Table 1; Fig. 3).

### Phenological traits

**Figure 4.**
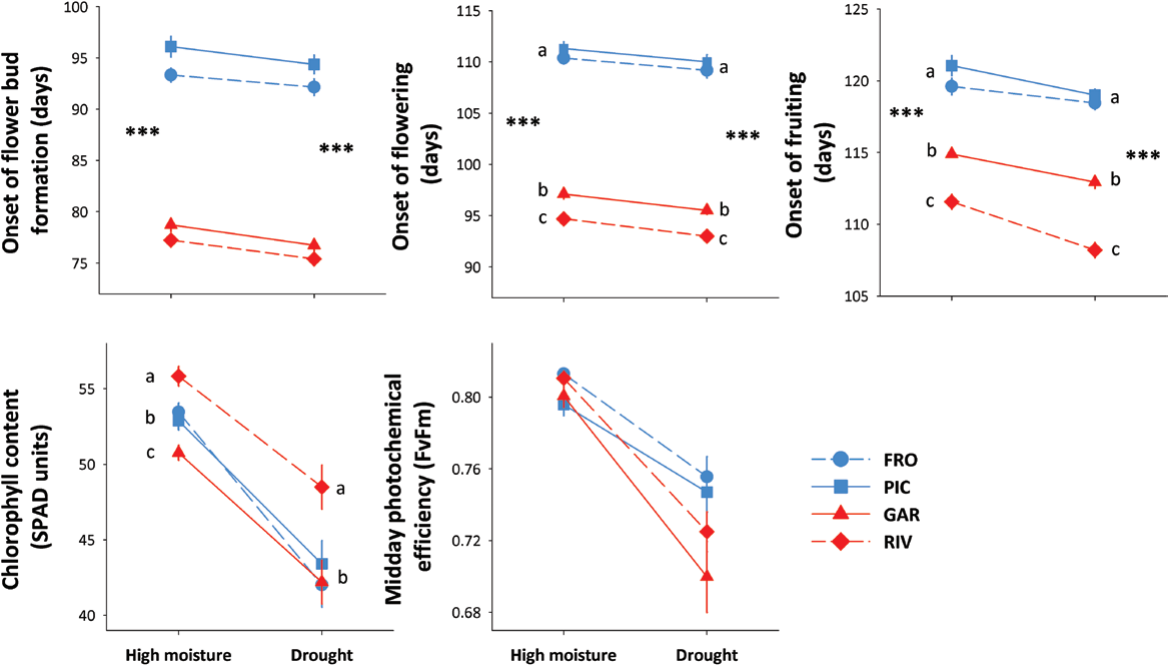
Population differences in phenological and physiological traits in two contrasting water availability treatments. Each line shows the norm of reaction of each of two northern (blue) and two southern (red) populations for a given trait. Observed population means ± SE are shown for 21 families per population, with three replicates per family and treatment. Significant regional differences in each watering treatment are shown with asterisks (no asterisk: not significant differences; **P < 0.01; ***P < 0.001). Different letters indicate significant differences among populations in mean trait values within each watering treatment, according to Tukey *post hoc* tests. The presence of both asterisks and letter indicates significant differentiation between regions and populations within regions. *N* = 504 plants for all traits. Population codes are those in Fig. 1.

There was a subtle but significant advance in the reproductive phenology of plants from all populations in the drought treatment (Table 1; Fig. 4). However, the greatest differences were found between plants from different regions, which was consistent in both watering treatments. On average, plants from the southern populations showed flower buds and open flowers ≈2 weeks before those from the northern populations, and advanced fruiting by 7.7 days. Significant differences were also found between populations within regions in the onset of flowering and fruiting, with plants from the southern population RIV flowering and fruiting significantly earlier than all other populations (Table 1; Fig. 4). No significant differences between regions or treatments were found for the duration of flowering onset **[see****Supporting Information—Fig. S5****]**.

### Physiological traits

Both measurements of photosynthetic activity, leaf chlorophyll content and the midday photochemical efficiency (*F*_v_/*F*_m_), were significantly lower in plants from all populations in the drought treatment (Table 1; Fig. 4). On average, plants in the drought treatment showed a 17.3 % and 8.5 % reduction on chlorophyll content and *F*_v_/*F*_m_, respectively, compared to plants in the high-moisture treatment (Fig. 4). No regional differences in the physiological traits were observed (Table 1). However, there was a significant difference at the population level in their chlorophyll content: in both treatments, plants from RIV showed higher chlorophyll content than any other population (Fig. 4).

### Leaf morphology and structure

**Figure 5.**
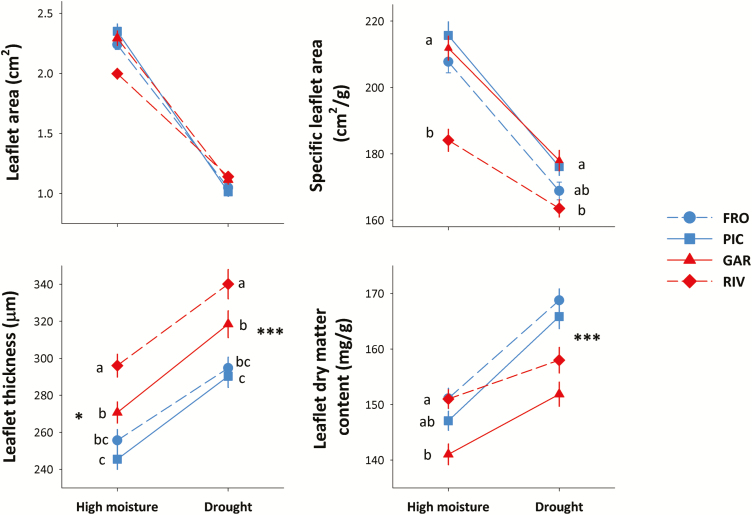
Population differences in leaflet morphology and structure in two contrasting water availability treatments. Each line shows the norm of reaction of each of two northern (blue) and two southern (red) populations for a given trait. Observed population means ± SE are shown for 21 families per population, with three replicates per family and treatment. Significant regional differences in each watering treatment are shown with asterisks (no asterisk: not significant differences; *P < 0.05; **P < 0.01; ***P < 0.001). Different letters indicate significant differences among populations in mean trait values within each watering treatment, according to Tukey *post hoc* tests. *N* = 504 plants for all traits. Population codes are those in Fig. 1.

Plants from all populations decreased leaflet area (2-fold reduction) and SLA (16.3 % decrease) in the drought treatment (Table 1; Fig. 5). No significant differences were found between regions in either trait, but SLA differed among populations: in both treatments, plants from the RIV population had the lowest SLA (Fig. 5). Furthermore, on the drought treatment, plants from all populations increased leaflet thickness (by an average 16 %) compared to those on the high-moisture treatment. In both treatments, plants from southern populations produced thicker leaflets than those from northern populations (Fig. 5). Significant differences were also found among populations, with plants from the RIV populations showing the thickest leaflets in both treatments. Finally, there was a 10 % increase in the LDMC of plants in the drought treatment. Differences between regions were observed only on the drought treatment (significant region × treatment; Table 1), with higher LDMC in plants from northern populations. In the high-moisture treatment, differences were found among populations, but not regions (Fig. 5).

### Reproductive traits

**Figure 6.**
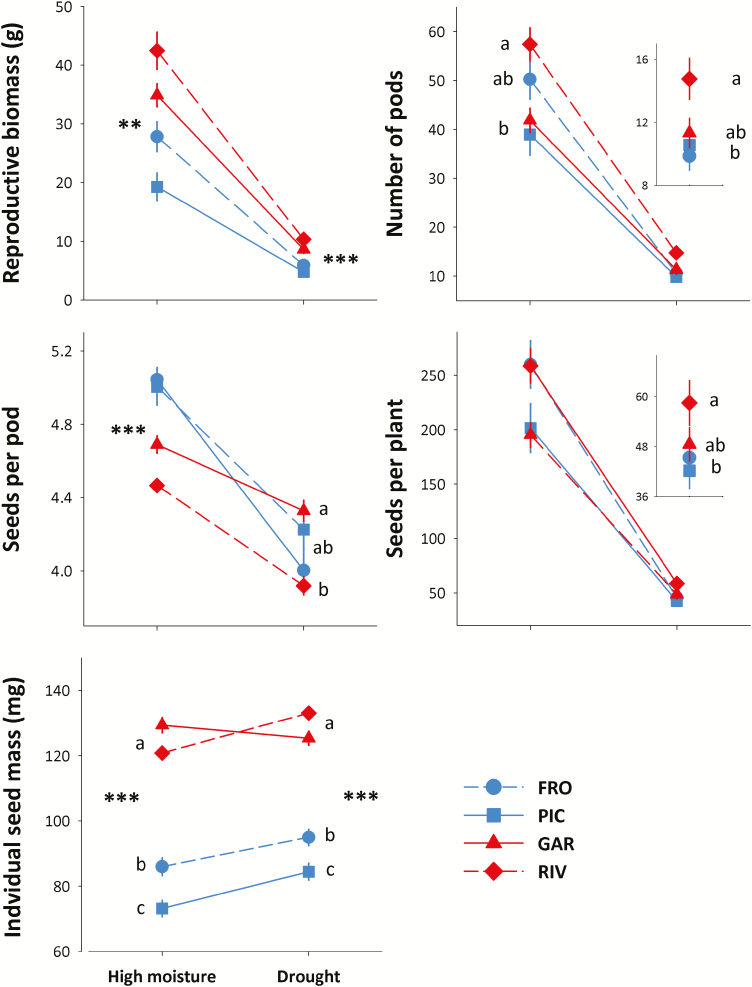
Population differences in reproductive traits in two contrasting water availability treatments. Each line shows the norm of reaction of each of two northern (blue) and two southern (red) populations for a given trait. Observed population means ± SE are shown for 21 families per population, with three replicates per family and treatment. Significant regional differences in each watering treatment are shown with asterisks (no asterisk: not significant differences; *P < 0.05; **P < 0.01; ***P < 0.001). Different letters indicate significant differences among populations in mean trait values within each watering treatment, according to Tukey *post hoc* tests. *N* = 504 plants for all traits. Population codes are those in Fig. 1.

Total reproductive biomass, number of pods and total number of seeds were c. four times greater in the high-moisture treatment compared to the drought treatment. Plants also produced fruits with significantly fewer seeds in the drought treatment, but increased the size of individual seeds (Table 1; Fig. 6). There were regional differences in the total reproductive biomass in both treatments, where plants from southern populations had higher reproductive biomass than those from northern populations. However, differences between regions were not found in the number of pods or the total number of seeds per plant (Table 1). In the high-moisture treatment, the higher number of fruits produced by plants from the RIV population was offset by the lower number of seeds per pod observed on both southern populations, overall leading to similar numbers of total seeds across populations in this treatment (Fig. 6). In the drought treatment, however, plants from the RIV population produced both a higher number of pods and of total seeds than the northern populations, despite showing the lowest number of seeds per pod of all populations. Finally, plants from the southern populations produced heavier seeds than those from the northern populations in both treatments (Fig. 6).

## Discussion

We found significant differences between regions that likely reflect adaptation to climatically distinct environmental conditions. This ecotypic differentiation was coupled with active plastic responses to varying water availability. Plasticity to drought was in most cases in the same direction as genetic differentiation, providing evidence of the adaptive value of the plastic change. Our results show that both mechanisms can generate adaptive phenotypic variation in *L. angustifolius*, and help identify potentially valuable genetic resources to incorporate into breeding programmes.

### Genetic differentiation between regions and populations

We found genetically based phenotypic differentiation between regions for traits of different functional categories. Southern populations had larger seeds, lower growth rates, earlier onset of reproduction and produced thicker leaves in both experimental treatments. Differentiation in several of these traits is congruent with adaptation of the southern populations to the conditions experienced at their sites of origin, as shown by their higher reproductive biomass in the drought treatment. For instance, the large differences observed in reproductive phenology between the southern—which started flowering >2 weeks earlier—and the northern populations may be an evolved response to the warmer (and drier) conditions during the southern reproductive period, that would result in a shorter reproductive phase. Indeed, previous studies on both wild (Milla *et al.* 2009) and domesticated (Dracup *et al.* 1998) plants of this species showed that early flowering and a later occurrence of the terminal summer drought had a positive effect on reproductive output (see also McKay *et al.* 2003; Méndez-Vigo *et al*. 2011; Matesanz *et al.* 2012).

Similarly, the thicker leaves and larger seeds of plants from the southern populations likely confer them an adaptive advantage to cope with the local conditions. Larger seeds may provide individuals with a head start for rapid seedling growth and a bet-hedging strategy in unpredictable and/or unfavourable environments (Leishman *et al.* 2000; Metz *et al.* 2010), and thicker leaves may avoid water loss during the warmer months of the southern growing period (Guerfel *et al.* 2009; Stagnari *et al.* 2018). Ecotypic differentiation in *L. angustifolius* has also been recently reported by Berger *et al.* (2017), who compared several cultivars and wild collections from diverse habitats across the species’ range. In line with our results, they found that ecotypes from drought-prone environments showed earlier phenology, larger seed size, higher vigour (early plant size) and reproductive output than collections from high-rainfall, cooler sites (see also Milla *et al.* 2009; Moncalvillo *et al.* 2019).

Adaptation to drought in the southern populations was not associated with lower performance in the high-moisture treatment. Plants from southern populations were able to successfully exploit the favourable, resource-rich environment, producing a similar or even higher number of pods, seeds per plant and total reproductive biomass than those from northern populations. This lack of reproductive trade-off in contrasting environments may be related, among other factors, to the levels of fine-grain environmental heterogeneity in the two contrasting regions (Sultan and Bazzaz 1993; Byers 2005; Matesanz *et al.* 2010; Baythavong 2011). Interannual variation based on the long-term climate of the study populations shows that southern populations experience more variable precipitation patterns (≈15 % more variable) than those from the north (data extracted from Karger *et al.* 2017). It is thus likely that plants from the south more often encounter temporal or spatial microsites similar to both of our experimental treatments, resulting in adaptive phenotypes in contrasting moisture conditions (see also Matesanz and Sultan 2013).

For several functional and reproductive traits, genetic differentiation was observed among populations rather than between regions. High population differentiation, despite the relatively short geographical distances between populations of the same region, suggests that factors other than regional climatic differences also drive genetic differentiation among populations in this species. These results may, at least partly, be explained by the mating system of our species or its dispersal ability (Loveless and Hamrick 1984). Highly selfing populations are expected to show strong population structure due to limited gene flow and the effects of genetic drift, as has been repeatedly shown for other predominantly selfers (see e.g. Heschel and Riginos 2005 and references therein; Volis 2011; Matesanz *et al.* 2012; Anderson *et al.* 2014; Matesanz *et al.* 2014). Further studies may help to determine whether within-region differentiation is the result of adaptation to a different environmental factor or simply reflects the effect of neutral evolutionary forces.

### Plasticity to drought in *L. angustifolius* populations

Drought affected all the functional traits measured in our study (Fig. 2). Some of these plastic changes, particularly those observed for growth and physiological traits such as lower growth rates, plant size and photochemical efficiency, clearly reflected the strong resource limitation imposed by the drought treatment. However, alongside these mostly unavoidable responses, plants from all populations also expressed active changes in the phenotypic expression of morphological, phenological and reproductive traits that were consistent with adaptive plasticity to drought. For instance, plants from the drought treatment showed an earlier onset of reproduction, in terms of both flower and pod production, compared to those on the high-moisture treatment, a response that has also been observed in cultivars of the study species when exposed to water shortage (Walker *et al.* 2011). This advanced reproductive phenology agrees with a drought-escape strategy, since plants showing delayed reproduction may not be able to complete their cycles before environmental conditions are too harsh (Cohen 1976; Fox 1990; Heschel and Riginos 2005; Franks *et al.* 2007; Munguía‐Rosas *et al.* 2011; Anderson *et al.* 2012; Ferris and Willis 2018). Differences in water availability before and during reproduction also altered the plant’s investment to reproductive units, as evidenced by the trade-off between the number of seeds per pod and individual seed size. Plants in drought conditions grew pods with fewer but larger seeds compared to plants that received ample water. Again, this is likely an adaptive response to drought, since increasing the size of individual propagules may boost seed germination and increase the chances of seedling establishment and survival when water is limiting (Leishman and Westoby 1994; Metz *et al.* 2010).

These plastic phenological and reproductive responses were also coupled with changes in leaf morphology and structure. Plants from all populations produced smaller leaves of lower specific leaf area, a well-documented response in drought conditions (Caruso *et al.* 2006; Capon *et al.* 2009; Lambrecht *et al.* 2017; Wellstein *et al.* 2017). Variations in SLA in response to water limitation may be due to changes in either one or both of its components, leaf thickness and leaf density (i.e. leaf dry matter content) (Witkowski and Lamont 1991; Wilson *et al.* 1999; Niinemets 2001; Guerfel *et al.* 2009; John *et al.* 2017). In our study, plants in the drought treatment increased leaf density and, to a larger extent, leaf thickness, together contributing to the decrease in specific leaf area. Higher leaf thickness is primarily related to an increase in the number of mesophyll cell layers, while higher leaf density is the result of thicker cuticle and/or cell walls and more tightly packed cells (Witkowski and Lamont 1991; Niinemets 2001; John *et al.* 2017). This coordinated response at the organ level likely reflects adaptive mechanisms to escape and tolerate drought, since lower SLA, higher density and thickness contribute to reduce the transpiration surface and increase leaf resistance to physical damage (Mediavilla *et al.* 2001; Guerfel *et al.* 2009; McLean *et al.* 2014; Deléglise *et al.* 2015; Stagnari *et al.* 2018).

A plastic response to an environmental factor in the same direction as genetic differentiation provides evidence of cogradient variation (Levins 1968; Conover and Schultz 1995; Eckhart *et al.* 2004; Crispo 2008; Kim and Donohue 2011; Ensing and Eckert 2019). Such coordinated variation also reflects the adaptive value of plasticity, since in the presence of cogradient variation plasticity produces phenotypic variation in the direction of the trait values also favoured by selection (Eckhart *et al.* 2004; Crispo 2008; Gonzalo-Turpin and Hazard 2009; Ensing and Eckert 2019 and references therein). In our study, we found cogradient variation in several instances. For example, drought induced both early phenology and the production of thicker leaves and larger seeds. Accordingly, populations from the south—experiencing drier conditions—precisely showed a faster onset of reproduction, higher leaf thickness and higher seed size. These results thus indicate that at least part of the plastic response observed in our study constitutes adaptive plasticity.

### Limitations of the study and future directions

The expression of genetic variation is environment-dependent. Therefore, it may be possible that the observed patterns of population differentiation were slightly affected by the environmental conditions experienced during the specific growing season when the experiment was performed (see Matesanz and Ramírez-Valiente 2019). Future experiments expanding different years and using the same genetic material would confirm whether such patterns of genetic variation are consistent across time (see also Exposito-Alonso *et al.* 2018). Furthermore, the inclusion of more populations from different regions would help assess whether genetic differentiation is shaped as clines along wider climatic gradients. Knowledge on the genetic composition of each population would inform of the role of intrapopulation processes on genetic differentiation and of potential responses to future selection. Finally, an exciting future research avenue in this system is testing the occurrence of adaptive transgenerational plasticity, i.e. the effects of the maternal environment on offspring development. The existence of this form of non-genetic inherited adaptation would constitute a yet not well-understood source of phenotypic variation.

## Conclusions

Despite strong site-of-origin differences in climatic conditions, all populations of our study species were able to plastically respond to contrasting levels of water availability. This high functional plasticity was coupled with adaptive differences among regions, providing evidence that both genetic differentiation and adaptive plasticity may be complementary rather than exclusive mechanisms contributing to plant adaptation to environmental variation (see also Sultan 1995; Kim and Donohue 2013; Matesanz and Valladares 2014; Palacio‐López *et al.* 2015). Furthermore, the patterns of differentiation observed in traits related to drought adaptation suggest that the southern populations may be a source of preadapted genotypes under the increase in aridity expected under climate change (Lázaro-Nogal *et al.* 2016), and highlight that certain populations of annual species may have the ability to cope with environmental change (see also Exposito-Alonso *et al.* 2018). Finally, our study also identified potentially valuable genetic resources to improve the currently small genetic pool of the domesticated material (Berger *et al.* 2013; Moncalvillo *et al.* 2019). The type of broadly adaptive genotypes present in some of our study populations, particularly those from the south, may be key to implement strategies aimed at breeding for adaptive plasticity, rather than for the production of canalized, not plastic genotypes (Milla *et al.* 2017; Matesanz and Milla 2018 and references therein).

## Supporting Information

The following additional information is available in the online version of this article—

**Table S1.** Geographical coordinates and climatic conditions of the sampled populations.

**Figure S1.** Population-level seed size.

**Figure S2.** Environmental conditions in the hoop greenhouse during seed germination and seedling growth.

**Figure S3.** Environmental conditions in the plasticity experiment.

**Figure S4.** Soil water content (%) in the two watering treatments.

**Figure S5.** Duration of the onset of flowering in each population and watering treatment.

plaa006_suppl_Supplementary_Files

## Data

All data and code are available at https://doi.org/10.6084/m9.figshare.11733495.v1.

## Sources of Funding

This work was funded by grants EVA (CGL2016-77377-R), GYPSEVOL (CGL2016-75566-P), and the Spanish Ramón y Cajal Programme of the Spanish Ministry of Economy and Competitiveness, and the European Union’s Horizon 2020 research and innovation programme under grant agreement No. 774271 (Farmer’s Pride project).

## Contributions by the Authors

J.M.I. and S.M. conceived and designed the study. M.L.R.T., M.R.-M., B.M., S.L.G.D., J.R. and S.M. established the experiment and collected all data. M.R.-M. and S.M. analysed the data. M.L.R.T., M.R.-M., J.M.I. and S.M. contributed to the discussion and interpretation of the results. S.M. wrote the manuscript, with input from all other authors.

## Conflict of Interest

None declared.
