# Supplementary material for: Plasticity to drought and ecotypic differentiation in populations of a crop wild relative
Source: AoB Plants. 2020 Feb 12;12(2):plaa006. doi: 10.1093/aobpla/plaa006 (PMC7065737; doi:10.1093/aobpla/plaa006)
Supplement: plaa006_suppl_Supplementary_Files [file plaa006_suppl_supplementary_files.pdf]

Table S1. Geographical coordinates and climatic conditions of the sampled populations. Altitude, mean, minimum and maximum annual temperature and precipitation are shown. Climatic data were obtained from CHELSA Climate (Karger *et al.* 2017). Data for the location of the plasticity experiment are also shown. The aridity index was calculated as P/T, where P is annual precipitation and T is mean annual temperature. SA = Semiarid; SH = Subhumid.

**Region**	**Population site**	**Coordinates**	**Altitude (m asl)**	**Mean annual temperature (ºC)**	**Minimum annual temperature (ºC)**	**Maximum annual temperature (ºC)**	**Precipitation (mm)**	**Index of aridity (Lang)**
N	Zafrón (FRO)	41.024192N; -6.028155W	836	12.59	7.76	17.42	506	40.19 (SH)
N	Zarapicos (PIC)	41.004358N; -5.813066W	822	12.79	7.95	17.63	472	36.90 (SH)
S	Rivera de la Lanchita (RIV)	38.351586N; -6.576084W	421	16.13	11.36	21.84	491	29.58 (SA)
S	La Garranchosa (GAR)	38.325735N; -6.433799W	356	16.60	10.88	21.38	531	32.92 (SA)
	CULTIVE Móstoles	40.334615N, -3º882168W	650	14.81	9.72	19.89	439	

Figure S1. Population-level seed size, based on family averages of 21 families and 16 seeds per family. Error bars represent 1se.

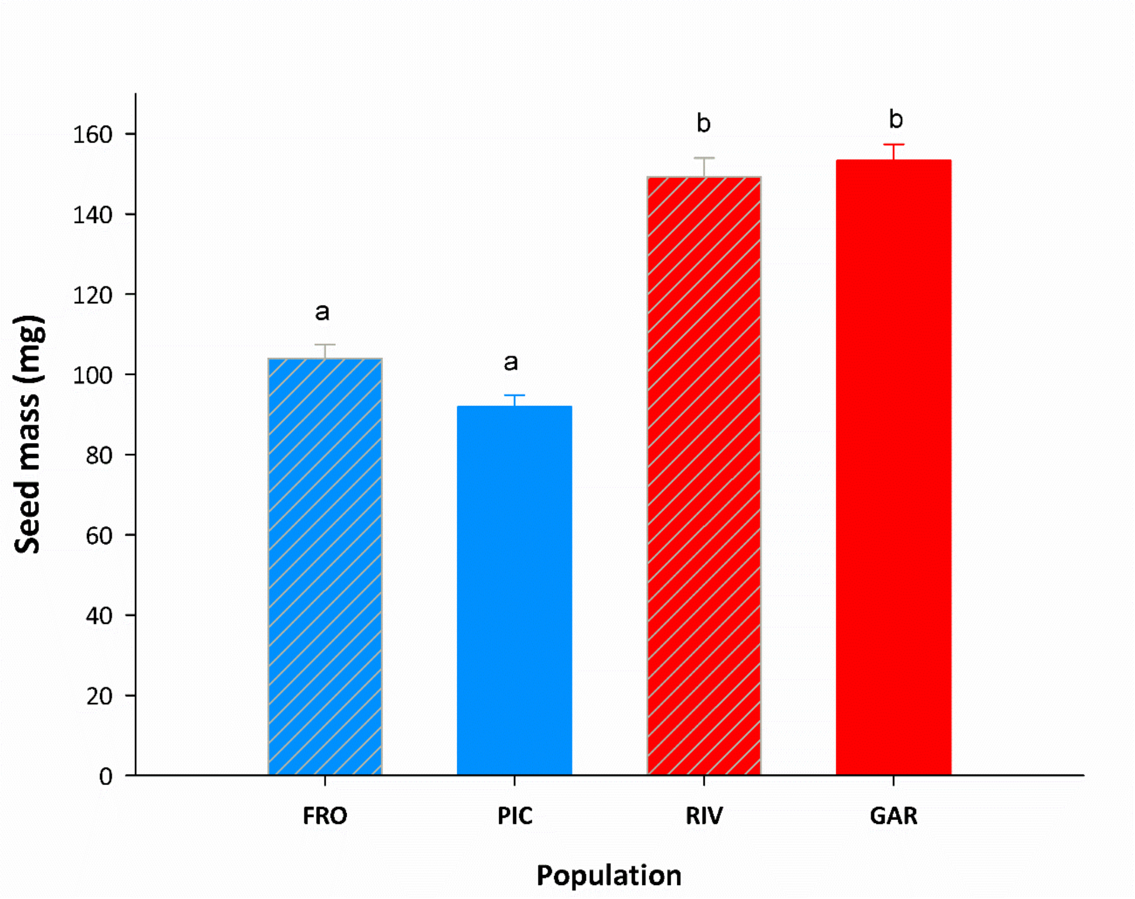

Figure S2. Environmental conditions in the hoop greenhouse during seed germination and seedling growth. Temperature (top) and relative humidity (bottom) were measured hourly with a HOBO Pro V2 data logger (Onset Co., Bourna, MA, USA). Dots represent daily averages.

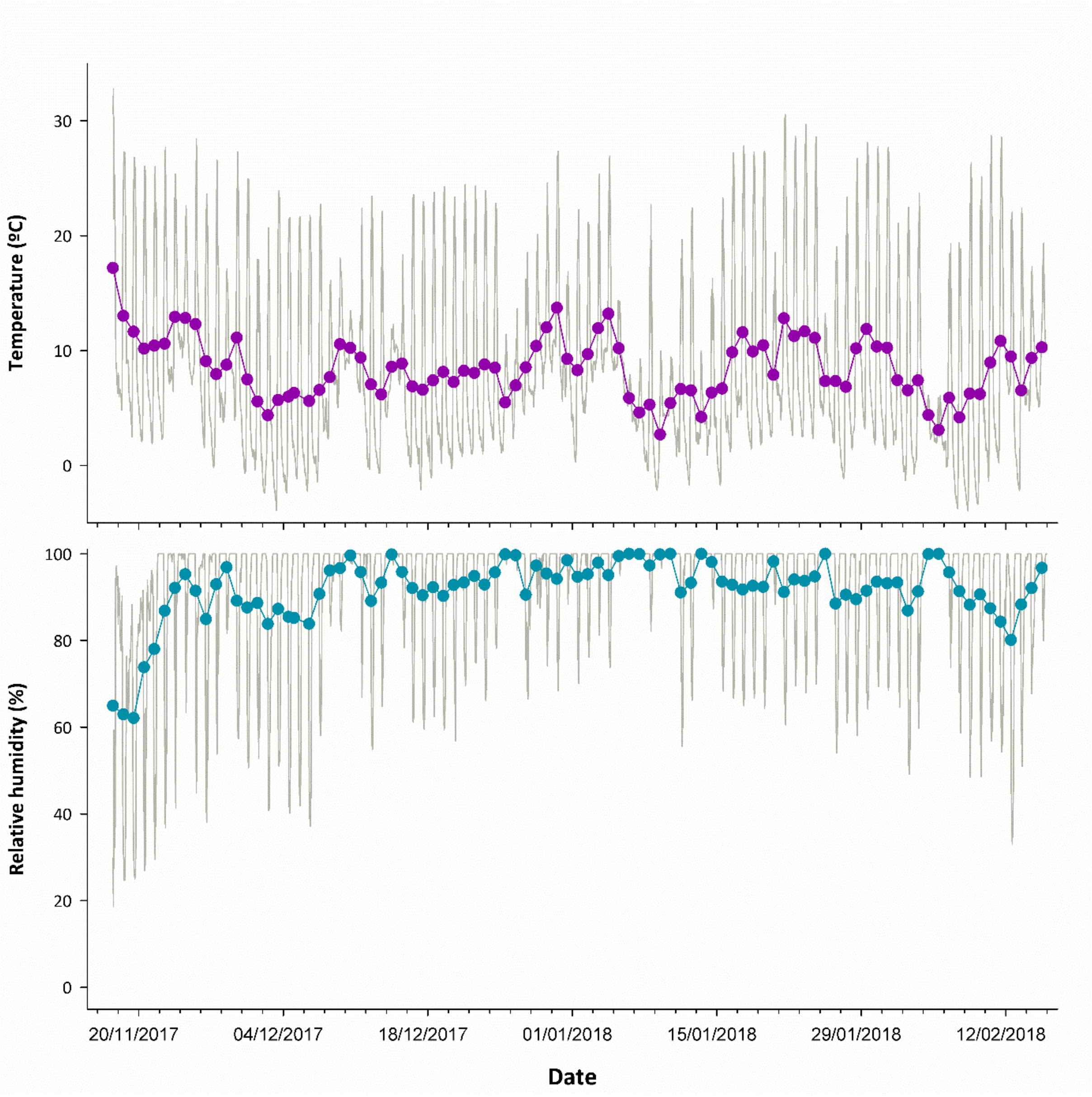

Figure S3. Environmental conditions in the plasticity experiment. Data were collected with a HOBO Micro station data logger (H21-USB, Onset Co., Bourna, MA, USA) that recorded photosynthetic active radiation (PAR, top panel), air temperature (middle panel) and relative humidity (lower panel) every 10 minutes throughout the experiment.

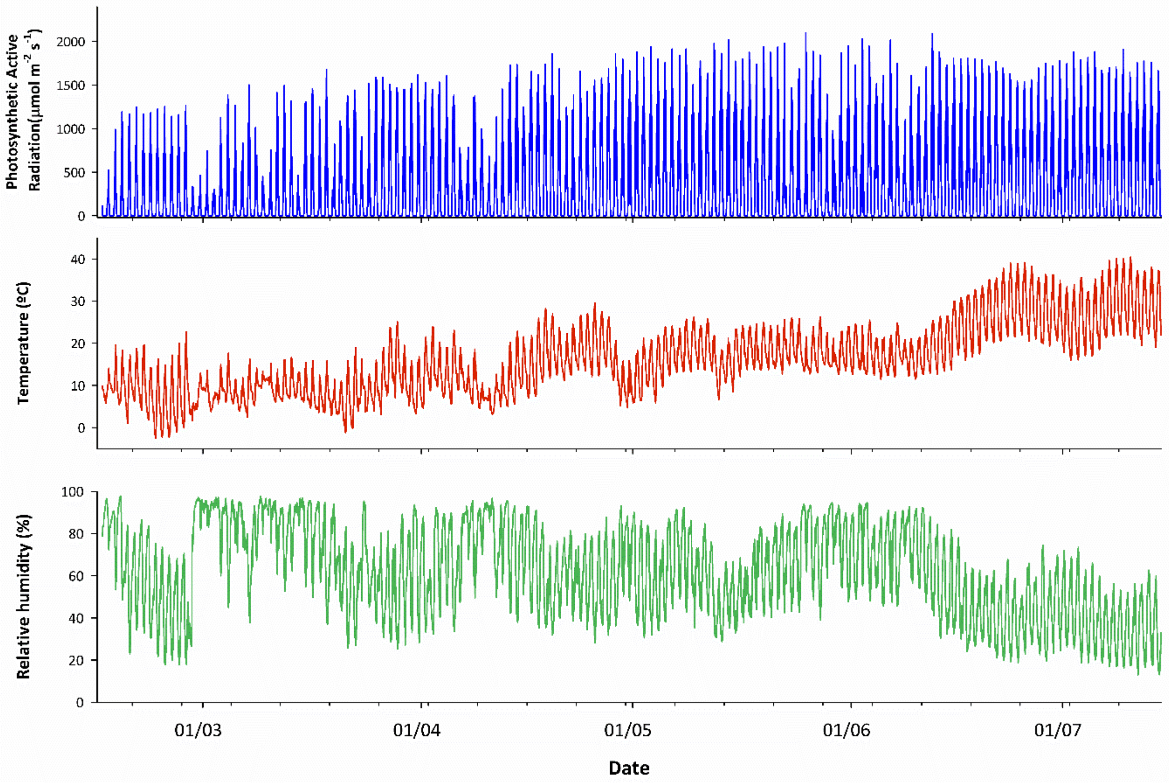

Figure S4. Soil water content (%) in the two watering treatments. Means ± s.e are shown at each treatment and timepoint. Plants in the high-moisture treatment were maintained at ≈100% of field capacity, and plants in the drought treatment were maintained at ≈35-40% of field capacity. Measurements were taken every 3-4 days in 15-20 pots per treatment using a HH2 Moisture Meter (Delta-T devices, Cambridge, UK). Water treatments were maintained until the last plant was removed from the experiment (early July, data not shown).

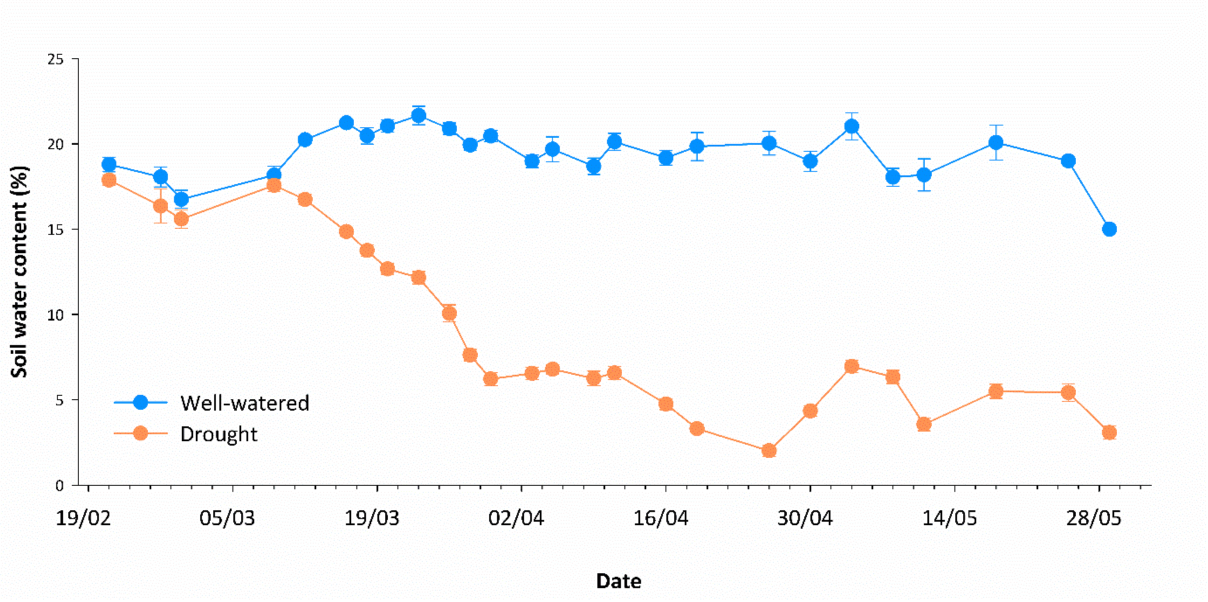

Figure S5. Duration of the onset of flowering in each population and watering treatment. No significant differences were found between regions (F = 2.98, P = 0.16) or treatments (F = 1.46, P = 0.29).

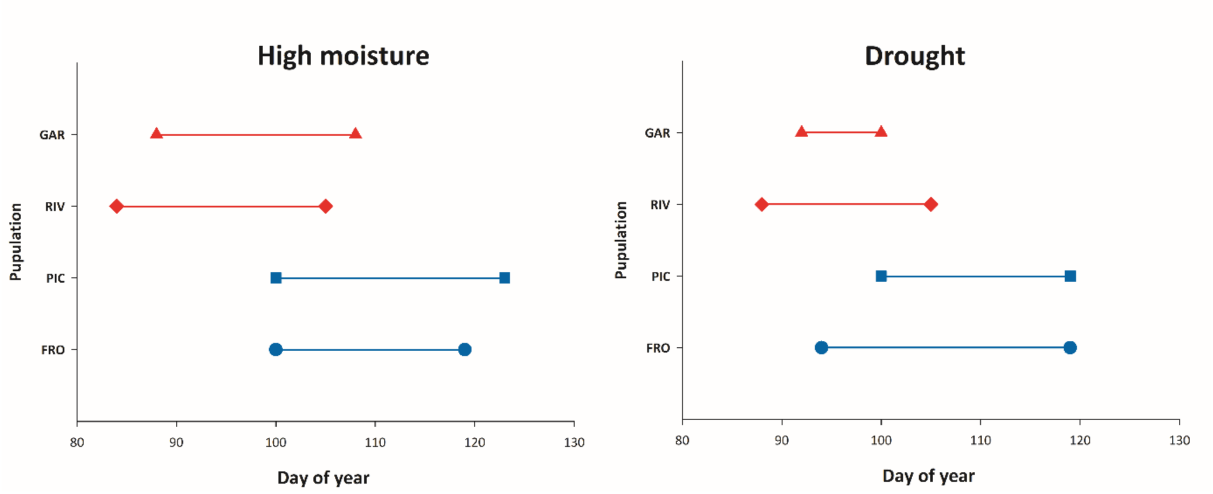

Karger DN, Conrad O, Böhner J, Kawohl T, Kreft H, Soria-Auza RW, Zimmermann NE, Linder HP, Kessler M. 2017. Climatologies at high resolution for the earth's land surface areas. *Scientific Data,* **4:** 170122.
